# Determinants and Differences of Township Hospital Efficiency among Chinese Provinces

**DOI:** 10.3390/ijerph16091601

**Published:** 2019-05-07

**Authors:** Bo Li, Muhammad Mohiuddin, Qian Liu

**Affiliations:** 1 International College of Business and Technology, Tianjin University of Technology, Tianjin 300384, China; great2011818@126.com; 2 School of Business and Economics, Thompson Rivers University, Kamloops, BC V2C 0C8, Canada; 3 School of Public Health, Tianjin Medical University, Tianjin 300070, China; liuqian2010@tmu.edu.cn

**Keywords:** efficiency, differences, township hospital

## Abstract

This study aimed to measure the efficiency and change in efficiency over time of township hospitals among Chinese provinces, to decompose the difference in efficiency between districts, and to study the correlations between the difference in efficiency and its determinants. Based on Chinese provincial panel data, the empirical analysis was established using data envelopment analysis (DEA), Malmquist index, Theil index decomposition method and Grey correlation analysis method. First, it was found that the township hospitals in most provinces were operating in an inefficient state, and the township hospitals in most provinces achieved gains in efficiency. Second, from 2003 to 2016 the shrinkage of the difference in provincial efficiency of township hospitals progressed slowly. Intra-regional difference is the main cause of the overall provincial efficiency difference of Chinese township hospitals, while inter-regional difference is the minor cause of the overall difference. Third, the correlation between the difference of overall provincial efficiency and the difference of economic development level is the highest among all the correlations, while other determinants rank second to seventh place in their degree of correlation with respect to the overall difference in provincial efficiency. Furthermore, the correlations between the intra-regional difference of provincial efficiency of Chinese township hospitals and its determinants vary tremendously across regions. Based on our findings, we can conclude, first, that efforts should be made to improve the overall provincial difference in efficiency of Chinese township hospitals, and enhance the utilization level of input resources, and to reduce resource waste. Second, in order to shrink the overall provincial efficiency of Chinese township hospitals, the most important measure that should be taken is to improve the economic development level in relatively backward provinces in order to lay a solid economic foundation for the improvement of efficiency and shrink the differences in efficiency between provinces. Third, more attention should be paid to the shrinkage of intra-regional efficiency differences in Chinese township hospitals, while the narrowing of inter-regional efficiency difference should not be ignored. For each region, it is necessary to recognize the difference in the relative importance of determinants, and to make development strategies according to local conditions so as to make full use of local characteristics and advantages.

## 1. Introduction

The aging of the population has led to an ever-increasing demand for medical and health care, which has made it increasingly contradictory to limited public health expenditure. As a result, improving the efficiency of the health care system has gradually become a core goal of the development of the health care system [1]. It is generally believed that medical and health care resources are not allocated in a balanced way. Related research pointed out that improving the service quality and controlling costs of primary health care institutions are the two main goals of health policies in many countries [2].

The health system serves a population of 1.3 billion in China, which is the World’s largest population [3]. However, more resources are concentrated on large-scale hospitals located in the cities. In rural areas, the medical and health care resources are in relative scarcity. In China, township hospitals are considered as the middle tier of a three-tiered rural medical system, which is organized at county, township and village levels [4]. At the intermediate level, township hospitals, providing acute and public health care [5], ensuring the linkage between village health clinics and county or above-level hospitals [6]. Furthermore, Chinese township hospitals, the main providers of primary health care [7], are the hubs of the rural tertiary health service system, dealing primarily with common disease management [8]. The primary health care system is the main component of China’s health system. Township hospitals play both a pivotal role in the delivery of health care services and a fundamental role in the primary health care system of China [9,10].

From 1950 to 1975, the efficient Chinese three-tier system of healthcare delivery has made great improvements to the population’s health [11,12]. However, according to the studies of some researchers, the economic transition has caused the deterioration of the three-tier rural healthcare system [13,14], and thus hampered the efficiency of township hospitals [15,16]. Furthermore, there exist apparent differences in the capacity of Chinese township hospitals. Even standardized clinical pathways cannot be implemented easily in township hospitals because of the considerable variation in the service capacities of rural institutions [17]. The differences will inevitably hamper the overall efficiency of Chinese township hospitals. In order to promote the service capacity of the Chinese primary medical and health care system, and to satisfy the demand for rural medical and health care services, and enhance the level of people’s health, it is necessary to improve Chinese township hospital efficiency. Therefore, it is of great theoretical and practical significance to explore effective ways of improving the efficiency of township hospitals in China. As can be seen from the existing studies, a considerable amount of literature has used data envelopment analysis method to study and evaluate the efficiency of Chinese township hospitals [18,19,20,21].

Compared to the previous literature, the contributions of this paper are as follows: (1) Using Chinese provincial panel data from 2003 to 2016, this paper measures the provincial efficiency and efficiency change of Chinese township hospitals using the DEA model and Malmquist index method, so as to provide a more complete picture to evaluate the operation of township hospitals among Chinese provinces; (2) this paper decomposes the efficiency difference using the Theil index decomposition method, so as to identify the structural sources of difference in efficiency among Chinese provinces; (3) this study further calculates the correlation and rank between differences of provincial efficiency and their determinants using the Grey correlation analysis method such that the relative importance of determinants of difference in efficiency can be measured. Through empirical analysis, the policy implications proposed by this paper will provide a reference for the coordinated development of township hospitals among provinces in China.

## 2. Literature Review

More and more researchers are focusing their attention on measuring the service efficiency of medical and health care institutions. Although much of the empirical research has focused on first-class hospitals [22], primary health care services are attracting more and more attention from researchers [23]. In fact, primary health care and health care systems need to play a key role in seeking an overall effective health care organization [24]. Therefore, the use of non-parametric methods, especially data envelopment analysis (DEA), has become common in empirical research because it can easily handle multiple dimensions of input and output primary health care indicators and is not easily affected by the problem of model setting, which is common for econometric models [25].

The measurement and evaluation of efficiency has always been a focus in the research of hospital management and health policy. The rate of hospitalizations due to ambulatory care sensitive conditions (ACSCs) is widely used as an indicator to measure the access and quality of primary care [26]. Furthermore, by developing quality indicators related to medical and health conditions, the access conditions and effectiveness of primary care at the hospital level can be monitored. A related study uses a completely non-parametric approach to estimate efficiency measures for primary care units and incorporates environmental factors into the consideration of efficiency improvement factors [27]. Health care systems in many countries are undergoing reform, including the United Kingdom, the United States and Denmark. In all countries, there is a great interest in how to improve the quality and efficiency of primary care [28]. In addition, there are several commonly used efficiency evaluation methods such as the ratio analysis method, comprehensive index method, rank sum ratio method, and data envelopment analysis. Data envelopment analysis is one of the relatively advanced evaluation methods [29].

At the same time, research on the influencing factors of the efficiency of medical and health service institutions has also become a focus of the research. As the level of urbanization increases, more people can effectively receive high-quality medical and health services. By improving the accessibility of health care services, it is beneficial to the efficiency of government expenditures and thus improves the level of primary health care services [30]. The greater the concentration of population per unit area in a region, the greater the economic effect of the scale of government public expenditure will be, resulting in an increase in the level of government public expenditure efficiency and thus improving the efficiency of primary medical services [31].

As a nation with a large number of rural residents, the construction of township hospitals as part of China’s new medical reform has been highly valued by the government at all levels. In particular, with the advancement of medical reform, the importance of rural primary health care has become increasingly prominent. In recent years, a large amount of health resources has been invested in this area. The extent to which these health resources are transformed into health care output has attracted many researchers’ attention. From the existing studies, a considerable amount of literature has used the data envelopment analysis method to study and evaluate the efficiency of China’s township health care institutions. Besides that, in studying the efficiency changes of township hospital construction projects, the total project investment funds and drug income as a percentage of business income can also be considered as input indicators [32]. 

As China has vastly different regions and many provinces, it is also one of the focuses of Chinese researchers to evaluate the service efficiency of provincial township health care institutions and their differences [33,34,35]. The findings of previous studies have laid the foundation for this paper. However, most of the existing literature has paid less attention to the change and difference of efficiency in Chinese township hospitals.

## 3. Methods

### 3.1. Data Envelopment Analysis and Malmquist Index

The method of data envelopment analysis (DEA) was first proposed by Charnes, Cooper and Rhodes as the CCR model [36]. The method entails the application of multiple input and output indicators and a linear program into the measurement of the technical efficiency for a production system. Generally speaking, the efficiency of a particular unit can be expressed as a ratio of the value of outputs to the value of inputs, where the efficiency of a unit must be less than or equal to one. The CCR model can be defined as follows:

(1) $$\begin{array}{c} \max_{\mu ,\nu }\theta =\mu_{1}y_{1o}+\ldots +\mu_{s}y_{so}\\ s.t.\;v_{1}x_{1o}+\ldots +v_{m}x_{mo}=1;\;\mu_{1}y_{1j}+\ldots +\mu_{s}y_{sj}\leq \nu_{1}x_{1j}+\ldots +\nu_{m}x_{mj},\;j=1,\ldots ,n\\ v_{1},v_{2},\ldots ,v_{m}\geq 0;\;\mu_{1},\mu_{2},\ldots ,\mu_{s}\geq 0 \end{array}$$

where θ is efficiency index, *x* represents input, *y* represents output, v represents input weight, μ represents output weight. 

By using other models, the overall technical efficiency can be further decomposed into the pure technical efficiency and scale efficiency. The aim of this paper is to deal with a relative efficiency comparison among provinces, and the focus here is the overall technical efficiency; therefore, following many related studies for China [37,38,39], the CCR model is chosen. Besides that, this paper focuses on how to improve the output effect of medical and health services under the established input conditions of township hospitals, so the output-oriented DEA model is selected.

In 1994, Fare et al. proposed the Malmquist index method based on the DEA method to measure the total factor of productivity change [40]. The method has been widely used in the analysis of technical efficiency and total factor productivity change studies.

The change in total productivity between two adjacent periods can be defined as follows:

(2) $$MI(x^{t},y^{t},x^{t+1},y^{t+1})={\left[\frac{D^{t}(x^{t+1},y^{t+1})}{D^{t}(x^{t},y^{t})}\times \frac{D^{t+1}(x^{t+1},y^{t+1})}{D^{t+1}(x^{t},y^{t})}\right]}^{1/2}$$

where *y* represents the output vector; *x* represents the input vector; *D^t^* (*x^t^, y^t^*) is defined as the output distance function, and *MI* (Malmquist index) measures the total productivity changes between period *t* and period *t*+1.

Using the Malmquist index method, the total factor productivity (TFP) change of the interested production system could be examined. Furthermore, the Malmquist Index can be further decomposed into two parts: the efficiency change (ECH), and the technological change (TCH). Therefore, MI can be expressed as follows:

(3) MI=ECH×TCH

### 3.2. Decomposition of Efficiency Difference

The Theil index calculation method is used as a measure to quantify the provincial efficiency difference, and Theil index decomposition method is used to analyze the structure of difference. The specific decomposition method is shown below:

(4) $$Theil=Theil_{W}+Theil_{B}$$

(5) $$Theil_{W}=\sum_{p=1}^{m}\left(\frac{n_{p}}{n}\frac{{\bar{e}}_{p}}{\bar{e}}\right)Theil_{p}$$

(6) $$Theil_{B}=\sum_{p=1}^{m}\frac{n_{p}}{n}\left(\frac{{\bar{e}}_{p}}{\bar{e}}\right)\ln\left(\frac{{\bar{e}}_{p}}{\bar{e}}\right)$$

where, m represents the number of regional groups, *n_p_*/*n* represents the proportion of the number of provinces of every region, ${\bar{e}}_{p}$/*e* represents the proportion of the index value of each region, *Theil_W_* and *Theil_B_* represent the intra-regional Theil index value and inter-regional Theil index value, respectively.

### 3.3. Grey Correlation Analysis 

Grey System theory was first proposed by Deng [41]. Since then it has become a preferred method to study and model systems [42]. The Grey correlation analysis method is an application of Grey System theory. Grey correlation analysis is a method of quantifying the correlation between factors of a system. According to this method, the parameters in the system are determined; the sequence is compared, and the original data are dimensionless. Furthermore, the point-to-interval distance method is used to calculate the difference (proximity), the correlation coefficient, and the degree of correlation. Finally, the degree of correlation between the judgment subsequence and the parent sequence is calculated. Grey correlation analysis can be used to effectively study the correlation between variables and analyze the relative importance of the variables. The detailed steps of the method can be expressed as follows:

First, given a raw data sequence ($x_{i}$): $x_{i}=\left(x_{i}\left(1\right),x_{i}\left(2\right),\ldots ,x_{i}\left(n\right)\right)$, conduct initial value treatment on raw data sequence ($x_{i}$) to obtain the normalized dimensionless sequence ($x_{i}^{\prime }$) as follows:

(7) $$x_{i}^{\prime }=x_{i}/x_{i}\left(1\right)=\left(1,x_{i}\left(2\right)/x_{i}\left(1\right),\ldots ,x_{i}\left(n\right)/x_{i}\left(1\right)\right)=\left(x_{i}^{\prime }\left(1\right),x_{i}^{\prime }\left(2\right),\ldots ,x_{i}^{\prime }\left(n\right)\right)$$

where *i* = 0, 1, 2, …, *m*

Second, obtain differential sequence as follow:

(8) $$\Delta_{i}\left(k\right)=\left|x_{0}^{\prime }\left(k\right)-x_{i}^{\prime }\left(k\right)\right|$$

Third, obtain the maximal proximity (*M*) and the minimal proximity (*m*) as follows:

(9) $$M=\max_{i}\;\max_{k}\Delta_{i}\left(k\right)$$

(10) $$m=\min_{i}\;\min_{k}\Delta_{i}\left(k\right)$$

where *i* = 1, 2, …, *m*; *k* = 1, 2, …, *n*

Fourth, solve the grey correlation coefficient.

(11) $$\gamma_{0i}\left(x_{0}\left(k\right),x_{i}\left(k\right)\right)=\frac{m+\xi M}{\Delta_{i}\left(K\right)+\xi M}$$

where $\gamma_{0i}$ is the grey correlation coefficient, ξ=0.5

Fifth, calculate the grey correlation of *x*_0_ and *x_i_* as follows:

(12) $$\gamma_{0i}=\frac{1}{n}\sum_{k=1}^{n}\gamma_{0i}\left(k\right)$$

where *i* = 0, 1, 2, …, *m.*

### 3.4. Data and Selection of Variables

Due to the consideration of data integrity and availability, in the measurement of Chinese township hospital efficiency, 29 provincial areas, including provinces, autonomous regions and municipalities directly under the central government, are treated as decision-making units for analysis. In the following parts of this paper, all of these provincial areas are named as provinces for simplicity. Beijing and Shanghai are not included as decision making units (DMUs) because there are no township hospital statistical values for these two cities.

The datasets used in this paper are obtained from the “China Health Statistical Yearbook” and “China Health and Family Planning Statistical Yearbook” from 2004 to 2017. The data of input and output variables selected and used in this paper are as follows. (1) Input indicator data: number of medical personnel (IN1); number of medical beds (IN2); number of township hospitals (IN3). (2) Output indicator data: number of outpatient visits (OUT1), because ACSCs is not used as a common acceptable statistical term in Chinese medical care system, the number of outpatient visits is used as an only available substitutive indicator; number of inpatients (OUT2); medical bed utilization rate (OUT3).

According to Table 1, it is clear to see the averages of input and output indicators of township hospital among major Chinese provinces: (1) the average number of medical personnel is 33,087. The maximum value (87,952) appears in Shandong, and the minimum value (2345) appears in Tibet; (2) the average number of medical beds in each province is 32,087. The maximum value (89,359) appears in Sichuan, and the minimum value (2291) appears in Ningxia; (3) the average number of township hospitals is 1336. Among the provinces, Sichuan has the maximum number of 4856, and Tianjin has the minimum number of only 169; (4) the average number of outpatient visits is 29,607,141. The maximum number of outpatient visits (80,329,663) is in Sichuan, and the minimum number (2551974) is in Qinghai; (5) the average number of inpatients is 1,046,449. The province with the maximum number of inpatients (3,568,686) is Sichuan, and the province with the minimum number (25,566) is Tibet; (6) the average medical bed utilization rate is 0.488. Among the provinces, Chongqing has the maximum rate of 0.648, and Tibet has the minimum rate of 0.298.

To analyze the structure of provincial efficiency difference of Chinese township hospitals, this paper uses eastern-central-western regional division. In the eastern region, there are Tianjin, Hebei, Liaoning, Jiangsu, Zhejiang, Fujian, Shandong, Guangdong, Hainan. In the central region, there are Shanxi, Jilin, Heilongjiang, Anhui, Jiangxi, Henan, Hubei, Hunan. In the western region, there are Inner-Mongolia, Guangxi, Chongqing, Sichuan, Guizhou, Yunnan, Tibet, Shaanxi, Gansu, Qinghai, Ningxia, Xinjiang. The regional division is based on the method provided in “China’s Health and Family Planning Statistical Yearbook 2013” [43].

In order to perform Grey correlation analysis between township hospital efficiency difference and a set of determinants at provincial level, the following data sequence has been considered: (1) the Theil index of provincial efficiency scores of Chinese township hospitals (x_0_); (2) Theil index of provincial proportion of medical technical personnel of the total number of medical personnel (x_1_); (3) Theil index of provincial proportion of medical managerial personnel of the total number of medical personnel (x_2_); (4) Theil index of provincial proportion of licensed (assistant) doctor in the total number of medical personnel (x_3_); (5) Theil index of provincial average years of schooling of population (x_4_); (6) Theil index of provincial proportion of medical and health expenditure in the provincial public financial expenditure (x_5_); (7) Theil index of provincial proportion of health care and medical services expenditure in per capita consumption expenditure of rural households by region (x_6_); (8) Theil index of provincial per capita real gross domestic product(GDP) (x_7_).

## 4. Empirical Results

### 4.1. Measurement of Efficiency and Change

The data envelopment analysis method is used to calculate the provincial health care service efficiency of Chinese township hospitals in 29 provinces of China from 2003 to 2016. The results are shown in Table 2. Due to space limitation, only the efficiency calculation results of 2003, 2005, 2007, 2009, 2011, 2013, 2015, 2016 and average by year are listed. As can be seen in the table, there exist apparent differences in the average efficiencies of township hospitals in the provinces of China. The township hospitals in most provinces were operating in an inefficient state. The top five provinces that have the highest average efficiencies are Tianjin and Ningxia. Furthermore, Inner-Mongolia, Shanxi and Jilin are the bottom three provinces that have the lowest average efficiencies.

In order to analyze the changing trend of provincial efficiency of Chinese township hospitals, the calculation method of Malmquist index is adopted. Using the method, this paper further measures the changes of efficiency of township hospitals in each province 2004 to 2016. The results are shown in Table 3.

As can be seen from the table, the average provincial efficiency of Chinese township hospitals for most provinces increased from 2004 to 2016. The average efficiencies of only two provinces, Fujian and Guangdong, decreased during the time period. Besides that, the average efficiencies of township hospitals in Tianjin and Ningxia remained unchanged because the township hospitals in these two provinces were in the efficient state all the time during the time period.

Overall, from 2004 to 2016, the township hospitals in most provinces achieved gains in efficiency, which was conducive to the promotion of provincial total factor productivity for township hospitals in China. Meanwhile, it is also clear that there still exist apparent efficiency differences in township hospitals among provinces, which is a problem that has adverse effects for the improvement of overall efficiency of Chinese township hospitals.

### 4.2. Decomposition of Efficiency Difference

**Figure 1:**
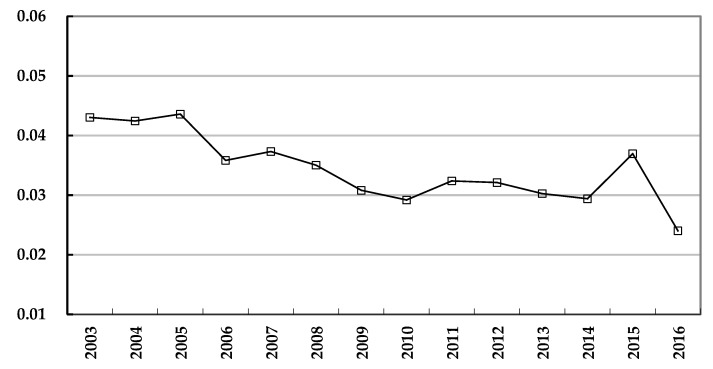
Theil index of provincial efficiency difference of Chinese township hospitals.

Through the results of provincial township hospital efficiency and efficiency change, it is clear that there exist apparent differences among provinces and regions. Therefore, this paper uses the Theil index to quantifiably measure the differences from 2003 to 2016, as shown in Figure 1.

As shown in the figure, the changing trend of the Theil index of provincial township hospital efficiency reflects the variation and tendency of efficiency difference change. It can be found that the Theil index was 0.04304 in 2003, and it showed a downward trend decreasing to 0.02402 in 2016, with fluctuations during the time period. Therefore, it is clear that from 2003 to 2016, the decline is only about 44.2% than the difference in 2003, which is a relatively slow shrinkage of the difference in provincial efficiency of township hospitals.

In order to find more effective ways of reducing provincial efficiency difference, it is necessary to find an appropriate way to quantifiably decompose the Theil index to study the structure of the difference, which would be helpful in finding the solution of the problem. Using the Theil index decomposition method, the results for 2003 to 2016 are shown in Table 4.

(1) Intra-regional difference is the main cause of the overall provincial efficiency difference of Chinese township hospitals, while the inter-regional difference is the minor cause in the overall difference. On average, during the time period from 2003 to 2016, the proportion of the sum of intra-regional differences was 87.0%, while the proportion of inter-regional difference was 13.0%. From the perspective of the proportions year by year, the proportions changed steadily. In 2003, the sum of proportions of intra-regional differences was 74.1%, while the proportion of the inter-regional difference was 25.9%. Although there were minor fluctuations, from 2003 to 2016, the sum of proportions of intra -regional differences increased to 98.1% in 2016, while the proportion of the inter-regional difference decreased to 1.9%.

(2) From the perspective of intra-regional difference for each region, the trends of the differences within the eastern, central and western regions of China were different year by year. Among them, although there were fluctuations during the time period, the proportion of intra-regional difference in eastern China in the overall difference decreased from 17.8% in 2003 to 11.0% in 2016, while the intra-regional difference in central China increased from 28% in 2003 to 51.0% in 2016, and the intra-regional difference in western China increased from 28.3% in 2003 to 36.0% in 2016. Furthermore, the proportion of intra-regional difference in central and western regions took the first place alternatively, while, the proportion of intra-regional difference in eastern region was the lowest through the time period. Except for 2004, 2005, 2006, 2014, 2015 and 2016, the proportion of intra-regional difference in western China was higher than the proportions of other regions during the time period. The proportion of intra-regional difference in central China took the second place in most years, but took the first place in 2004, 2005, 2006, 2014, 2015 and 2016. The intra-regional difference in eastern China was lower than the other two regions from 2003 to 2016, therefore it is relatively unimportant to the overall provincial efficiency difference of Chinese township hospitals.

### 4.3. Determinants of Provincial Efficiency Difference of Chinese Township Hospitals

To find effective solutions to the shrinkage of the overall efficiency difference, it is necessary to further investigate the relative importance of the determinants for the difference. This paper uses the Grey correlation analysis model to study the correlation between overall provincial efficiency difference of Chinese township hospitals (x_0_) and the determinants of difference including: (1) difference of medical technical personnel proportion (x_1_); (2) difference of medical managerial personnel proportion (x_2_); (3) difference of licensed doctor and assistant doctor proportion (x_3_); (4) difference of provincial average years of schooling (x_4_); (5) difference of public medical and health expenditure proportion (x_5_); (6) difference of rural household health care and medical services expenditure proportion (x_6_); (7) difference of economic development level (x_7_).

Compared to the conventional statistical coefficients, Grey correlation analysis has much less strict requirements on the sample size. For each year, only one value of each Theil index could be obtained. Therefore, Grey correlation analysis is more appropriate for this paper than other conventional statistical methods.

According to the steps of the Grey relational analysis method, the Grey correlations between provincial efficiency difference of Chinese township hospitals and the determinants can be obtained. As shown in Table 5, initial value treatment is conducted on raw data sequences.

Based on the results of initial value treatment, dimensionless differential sequences are calculated as shown in Table 6.

According to the difference sequence in the table, the following values could be obtained:

M=1.1296,m=0

Based on the results of difference sequence in the table 6, the grey correlation table are calculated as shown in Table 7.

Furthermore, the Grey correlations between provincial efficiency differences of Chinese township hospitals (sequence x_0_) and the determinants of efficiency difference (sequence x_1_–x_7_) could be solved. The results are shown in Table 8.

According to the correlation results in Table 8, among the determinants, the degree of correlation between x_0_ and x_7_ (difference of economic development level) is the highest among all the correlations. Then, x_1_ (difference of medical technical personnel proportion), x_4_ (difference of provincial average years of schooling), x_5_ (difference of public medical and health expenditure proportion), x_6_ (difference of rural household health care and medical services expenditure proportion), x_2_ (difference of medical managerial personnel proportion), x_3_ (difference of licensed doctor and assistant doctor proportion) take the second to seventh place in their degree of correlation with x_0_ (overall provincial efficiency difference of Chinese township hospitals).

Furthermore, because the decomposition results of the Theil index reveal the structural causes of the provincial efficiency difference of Chinese township hospitals, i.e., the intra-regional difference is the major cause of the overall difference, this paper further examines the Grey correlations between intra-regional difference of provincial efficiency difference of Chinese township hospitals and determinants within each of the eastern, central and western regions of China. The results are shown in Table 9.

According to the results in Table 9, it is clear to see that the correlations between the intra-regional difference of provincial efficiency of Chinese township hospitals and determinants vary tremendously across different regions in China.

First of all, for the eastern region, x_4_ (difference of provincial average years of schooling) has the highest correlation with x_0_. The remaining determinants, including x_7_ (difference of economic development level), x_5_ (difference of public medical and health expenditure proportion), x_3_ (difference of licensed doctor and assistant doctor proportion), x_1_ (difference of medical technical personnel proportion), x_2_ (difference of medical managerial personnel proportion), x_6_ (difference of rural household health care and medical services expenditure proportion), rank from the second position to the seventh position with respect to their correlation with x_0_.

Secondly, for the central region, x_7_ (difference of economic development level) has the highest correlation with x_0_. The remaining determinants, including x_3_ (difference of licensed doctor and assistant doctor proportion), x_1_ (difference of medical technical personnel proportion), x_6_ (difference of rural household health care and medical services expenditure proportion), x_5_ (difference of public medical and health expenditure proportion), x_4_ (difference of provincial average years of schooling), x_2_ (difference of medical managerial personnel proportion), rank from the second position to the seventh position with respect to their correlation with x_0_.

Finally, for the western region, x_6_ (difference of rural household health care and medical services expenditure proportion) has the highest correlation with x_0_. The remaining determinants, including x_2_ (difference of medical managerial personnel proportion), x_1_ (difference of medical technical personnel proportion), x_4_ (difference of provincial average years of schooling), x_5_ (difference of public medical and health expenditure proportion), x_7_ (difference of economic development level), x_3_ (difference of licensed doctor and assistant doctor proportion), rank from the second position to the seventh position with respect to their correlation with x_0_.

## 5. Discussions on Findings

Based on Chinese provincial panel data from 2003 to 2016, using the DEA model and Malmquist index, this paper measures the provincial efficiency and change in efficiency of Chinese township hospitals. Based on the measurement of efficiency, using the Theil index decomposition method, this paper measures and decomposes the provincial efficiency difference of Chinese township hospitals. Furthermore, based on the measurement and decomposition of overall provincial efficiency difference of Chinese township hospitals, using the Grey correlation analysis method, this paper studies the relationship between overall provincial efficiency difference of Chinese township hospitals and the determinants of difference, and further measures and investigates the intra-regional provincial efficiency difference of Chinese township hospitals, and the determinants within the eastern, central and western regions of China.

The empirical results are as follows. First, in general, the township hospitals in most provinces were operating in an inefficient state. From 2003 to 2016, the township hospitals in most provinces achieved the efficiency gains, which was conducive to the promotion of provincial total factor productivity for township hospitals in China. However, there still exist apparent efficiency differences of township hospitals among provinces.

Second, from 2003 to 2016, the decline of the Theil index of provincial township hospital efficiency is only about 44.2% lower than the difference in 2003, which is a relatively slow shrinkage of the difference in provincial efficiency of township hospitals. Intra-regional difference is the main cause of the overall provincial efficiency difference of Chinese township hospitals, while the inter-regional difference is the minor cause in the overall difference. Furthermore, during this time period, the proportion of intra-regional difference in central and western regions took the first place alternatively, while the proportion of intra-regional difference in the eastern region was the lowest

Third, compared to the conventional statistical coefficients, Grey correlation analysis has a much less strict requirement on the sample size. Therefore, it is more appropriate for this paper than other methods. For all 29 provinces, the correlation between the difference of overall provincial efficiency difference of Chinese township hospitals and the difference of economic development level is the highest among all the correlations. Further Grey correlation analysis for the eastern, central and western regions show that the correlations between the intra-regional difference of provincial efficiency of Chinese township hospitals and determinants vary tremendously across regions. 

Education takes the first place in the correlation degree in the eastern region; economic development takes the first place in the central region, and household healthcare expenditure proportion takes the first place in the western region. The economic and political implication behind these results is that due to socio-economic inequality among the eastern, central and western regions, the most important determinants of provincial efficiency difference of township hospitals for each region are also varied. The eastern region enjoys the highest level of economic development; therefore, the most important restrictive factor for equalizing the provincial efficiency difference of township hospitals is the gap of individual knowledge and education among provinces. The central region is mediocre with respect to economic development, which has become the biggest impediment in the realization of equality in the provincial efficiency of township hospitals. The western region is relatively lagging behind in both economic development and individual income; therefore, the difference in affordability of household medical care expense becomes the most important determinant of provincial difference in the efficiency of township hospitals in this region.

## 6. Conclusions

Our study focused on measuring the efficiency and change in efficiency over time of township hospitals among Chinese provinces, and decomposing the difference in efficiency between districts, and studying the correlations between the difference in efficiency and its determinants. We found that township hospitals in most provinces were operating, in general, in an inefficient state but improving. Intra-regional difference is the main cause of the overall provincial efficiency difference of Chinese township hospitals, while the inter-regional difference is the minor cause in the overall difference. Socio-economic differences are the most important determinants of provincial efficiency difference of township hospitals for each region. Our findings imply the following policy implications. 

First, efforts should be made to improve the overall provincial efficiency difference of Chinese township hospitals, to enhance the utilization level of input resources, and reduce resource wastes. Further measures should be taken to restructure the input-output patterns of township hospitals in each province, especially for relatively backward provinces that have relatively lower efficiencies, so as to improve the overall provincial efficiency of Chinese township hospitals.

Second, in order to shrink the overall provincial efficiency of Chinese township hospitals, the most important measure that should be taken is to improve the economic development level in relatively backward provinces in order to lay a solid economic foundation for the improvement of efficiency and shrink the differences in township hospital among provinces. For township hospitals in relatively backward provinces, it is necessary to increase the proportion of medical technical personnel in order to shrink the difference of professional levels among provinces. Local governments in relatively backward provinces should take measures to improve the level of education, increase public financial support for township hospitals and guide household expenditure to invest more on health care and medical services through public education, so as to shrink the differences among provinces. Furthermore, township hospitals in relatively backward provinces should not ignore the effects of increasing the proportion of licensed doctors and assistant doctors, and the proportion of managerial personnel in the total number of medical personnel.

Third, more attention should be paid to the shrinkage of intra-regional efficiency differences of Chinese township hospitals, while the narrowing of inter-regional efficiency difference should not be ignored. For each region, it is necessary to recognize the difference in the relative importance of determinants. For the eastern region, focus should be put on the shrinkage of differences in provincial education level, economic development, and public financial support for health care and medical services. For the central region, focus should be put on the shrinkage of differences in economic development, proportion of licensed doctor and assistant doctors and the proportion of medical technical personnel of the total number of medical personnel. For the western region, focus should be put on the shrinkage of differences in rural household health care and medical services expenditure, and the proportion of managerial personnel and proportion of medical technical personnel in the total number of medical personnel. Local governments should make development strategies according to local conditions, so as to make full use of local characteristics and advantages. Besides that, effective measures should also be taken to activate inter-regional positive interactions and facilitate communication between regions, so as to guide the comprehensive development of Chinese township hospitals.

Furthermore, this paper could include that the indicators and performance of township hospital conditions should be extended according to different categories of illness or health in order to further investigate the difference and determinants of provincial efficiency of Chinese township hospitals. 

## Figures and Tables

**Table 1:** Average of Input and Output Variables by Provinces (2003–2016).

No.	Province	IN1	IN2	IN3	OUT1	OUT2	OUT3
1	Tianjin	4304	3253	169	5,709,230	93,767	0.465
2	Hebei	42,381	51,613	2052	38,052,196	1,318,891	0.477
3	Shanxi	23,115	25,757	1386	13,538,673	374,279	0.381
4	Inner-Mongolia	17,703	15,874	1348	11,553,637	326,647	0.372
5	Liaoning	19,209	25,335	1011	13,067,216	571,884	0.414
6	Jilin	19,575	15,389	785	8,924,297	259,827	0.299
7	Heilongjiang	18,606	17,152	960	9,238,032	493,656	0.481
8	Jiangsu	62,729	54,292	1270	67,745,825	1,362,398	0.532
9	Zhejiang	39,603	17,747	1609	66,706,220	271,238	0.366
10	Anhui	44,058	45,488	1651	39,197,999	1,501,634	0.530
11	Fujian	22,868	23,261	890	21,247,725	1,003,161	0.521
12	Jiangxi	33,834	31,531	1557	24,133,911	1,740,297	0.637
13	Shandong	87,952	79,805	1655	61,731,640	2,167,761	0.485
14	Henan	74,541	75,782	2071	68,857,241	2,521,138	0.556
15	Hubei	59,288	47,226	1146	42,125,559	1,478,512	0.594
16	Hunan	62,645	62,036	2351	37,329,805	2,339,213	0.591
17	Guangdong	62,876	45,492	1292	69,463,572	1,658,462	0.531
18	Guangxi	41,749	39,890	1277	38,481,307	1,950,776	0.582
19	Hainan	6773	5064	304	7,893,964	104,146	0.312
20	Chongqing	24,154	27,734	1022	23,727,277	1,127,293	0.648
21	Sichuan	71,305	89,359	4856	80,329,663	3,568,686	0.585
22	Guizhou	22,307	26,282	1438	18,426,504	1,260,365	0.548
23	Yunnan	24,059	33,585	1406	32,225,007	1,036,330	0.494
24	Tibet	2345	2649	671	2,916,278	25,566	0.298
25	Shaanxi	28,601	25,318	1678	18,683,644	535,670	0.403
26	Gansu	19,604	19,175	1370	17,202,453	463,310	0.467
27	Qinghai	3476	3097	403	2,551,974	105,652	0.503
28	Ningxia	3246	2291	239	4977634	48,550	0.459
29	Xinjiang	16,618	19,049	888	12568620	637916	0.628
-	Average	33,087	32,087	1336	29607141	1046449	0.488

**Table 2:** Provincial efficiency of Chinese township hospitals (2003–2016).

No.	Province	2003	2005	2007	2009	2011	2013	2015	2016	Average
1	Tianjin	1.000	1.000	1.000	1.000	1.000	1.000	1.000	1.000	1.000
2	Hebei	0.754	0.612	0.728	0.724	0.726	0.661	0.718	0.793	0.704
3	Shanxi	0.354	0.337	0.382	0.418	0.475	0.489	0.408	0.554	0.426
4	Inner-Mongolia	0.459	0.514	0.511	0.506	0.511	0.513	0.446	0.536	0.498
5	Liaoning	0.523	0.642	0.520	0.542	0.617	0.619	0.569	0.717	0.592
6	Jilin	0.347	0.440	0.458	0.437	0.415	0.338	0.293	0.343	0.390
7	Heilongjiang	0.420	0.449	0.530	0.621	0.482	0.610	0.679	0.740	0.556
8	Jiangsu	0.814	0.810	0.824	0.909	1.000	1.000	1.000	1.000	0.912
9	Zhejiang	0.960	1.000	1.000	1.000	1.000	1.000	1.000	1.000	0.997
10	Anhui	0.698	0.806	0.684	0.734	0.797	0.821	0.879	0.873	0.777
11	Fujian	1.000	1.000	1.000	0.885	0.908	0.756	0.744	0.765	0.896
12	Jiangxi	0.946	1.000	1.000	1.000	1.000	0.957	1.000	1.000	0.993
13	Shandong	0.731	0.724	0.861	0.868	1.000	0.886	0.800	0.809	0.838
14	Henan	0.642	0.734	0.929	0.817	0.961	0.883	1.000	1.000	0.860
15	Hubei	0.544	0.580	0.640	0.800	0.958	0.957	1.000	1.000	0.803
16	Hunan	0.500	0.551	0.565	0.674	0.886	0.827	0.953	0.983	0.727
17	Guangdong	1.000	1.000	1.000	1.000	1.000	0.959	0.960	0.978	0.989
18	Guangxi	0.846	0.917	0.993	1.000	1.000	1.000	1.000	1.000	0.970
19	Hainan	0.647	0.707	0.717	0.745	0.883	0.815	0.730	0.782	0.757
20	Chongqing	0.798	0.857	0.951	1.000	1.000	1.000	1.000	1.000	0.953
21	Sichuan	0.859	0.966	0.962	0.910	0.929	0.927	0.956	1.000	0.942
22	Guizhou	0.615	0.747	0.930	1.000	1.000	1.000	0.798	0.743	0.872
23	Yunnan	0.725	0.837	0.981	1.000	1.000	1.000	0.985	1.000	0.944
24	Tibet	1.000	1.000	1.000	0.873	0.769	0.909	0.543	0.816	0.859
25	Shaanxi	0.509	0.557	0.538	0.504	0.466	0.499	0.584	0.591	0.530
26	Gansu	0.728	0.750	0.745	0.664	0.580	0.610	0.643	0.725	0.685
27	Qinghai	0.681	1.000	1.000	1.000	1.000	1.000	0.931	1.000	0.972
28	Ningxia	1.000	1.000	1.000	1.000	1.000	1.000	1.000	1.000	1.000
29	Xinjiang	0.533	0.673	0.637	0.607	0.767	0.793	0.958	1.000	0.724
-	Average	0.711	0.766	0.796	0.801	0.832	0.822	0.813	0.853	0.799

**Table 3:** Provincial efficiency change through Malmquist index of Chinese township hospitals (2004–2016).

No.	Province	2004	2006	2008	2010	2012	2014	2016	Average
1	Tianjin	1.000	1.000	1.000	1.000	1.000	1.000	1.000	1.000
2	Hebei	0.872	1.059	1.058	0.969	0.938	1.037	1.104	1.004
3	Shanxi	1.017	1.064	1.092	1.039	1.022	1.014	1.357	1.035
4	Inner-Mongolia	1.032	0.959	0.983	1.019	0.925	1.016	1.201	1.012
5	Liaoning	1.066	0.860	1.142	1.057	0.995	1.051	1.259	1.025
6	Jilin	0.997	0.951	0.984	1.018	0.945	1.022	1.168	0.999
7	Heilongjiang	0.955	0.958	1.099	1.100	1.110	1.009	1.089	1.044
8	Jiangsu	1.053	1.004	1.044	0.975	1.000	1.000	1.000	1.016
9	Zhejiang	1.041	1.000	1.000	1.000	1.000	1.000	1.000	1.003
10	Anhui	0.968	0.809	1.061	1.093	1.078	1.062	0.994	1.017
11	Fujian	1.000	1.000	1.000	0.962	0.951	1.019	1.028	0.980
12	Jiangxi	1.050	1.000	1.000	1.000	1.000	1.045	1.000	1.004
13	Shandong	0.917	1.028	1.010	1.046	1.000	0.966	1.011	1.008
14	Henan	0.991	1.027	1.002	1.033	0.986	1.089	1.000	1.035
15	Hubei	1.096	1.072	1.095	1.061	1.044	1.045	1.000	1.048
16	Hunan	1.005	1.118	1.110	1.049	0.968	1.129	1.031	1.053
17	Guangdong	1.000	1.000	1.000	1.000	0.988	0.999	1.019	0.998
18	Guangxi	1.031	1.040	1.007	1.000	1.000	1.000	1.000	1.013
19	Hainan	0.983	1.070	0.980	1.176	0.913	0.978	1.072	1.015
20	Chongqing	1.063	1.057	1.052	0.987	1.000	1.000	1.000	1.017
21	Sichuan	1.066	0.983	1.039	0.965	1.013	1.079	1.047	1.012
22	Guizhou	1.053	0.984	1.076	1.000	1.000	1.000	0.932	1.015
23	Yunnan	1.126	1.043	1.019	1.000	1.000	1.000	1.015	1.025
24	Tibet	0.617	1.000	1.000	0.950	1.205	0.813	1.503	0.984
25	Shaanxi	1.107	0.975	1.051	0.925	1.059	1.091	1.011	1.011
26	Gansu	1.085	1.036	1.034	0.860	1.052	1.021	1.127	1.000
27	Qinghai	1.469	1.000	1.000	1.000	1.000	1.000	1.075	1.030
28	Ningxia	1.000	1.000	1.000	1.000	1.000	1.000	1.000	1.000
29	Xinjiang	0.997	0.930	1.016	1.138	0.935	1.208	1.043	1.050
-	Average	1.023	1.001	1.033	1.015	1.004	1.024	1.072	1.016

**Table 4:** Contribution decomposition of Theil index (2003–2016).

Year	Intra-Regional			Sum of Intra-Regional	Inter-Regional
	Eastern	Central	Western		
2003	17.8%	28.0%	28.3%	74.1%	25.9%
2004	19.3%	29.5%	27.5%	76.3%	23.7%
2005	17.2%	34.1%	26.2%	77.5%	22.5%
2006	17.8%	29.7%	29.1%	76.6%	23.4%
2007	18.0%	30.8%	31.7%	80.5%	19.5%
2008	15.0%	30.4%	36.2%	81.6%	18.4%
2009	16.9%	28.1%	42.1%	87.1%	12.9%
2010	15.2%	29.5%	46.5%	91.1%	8.9%
2011	12.1%	39.3%	40.5%	91.9%	8.1%
2012	14.2%	39.3%	41.2%	94.6%	5.4%
2013	15.0%	38.6%	39.3%	92.9%	7.1%
2014	12.8%	45.8%	37.8%	96.5%	3.5%
2015	14.2%	48.6%	36.1%	98.9%	1.1%
2016	11.0%	51.0%	36.0%	98.1%	1.9%
**Average**	**15.5%**	**35.9%**	**35.6%**	**87.0%**	**13.0%**

**Table 5:** Results of initial value treatment for Grey correlation analysis (2003–2016).

Seq.	2003	2004	2005	2006	2007	2008	2009	2010	2011	2012	2013	2014	2015	2016
x_0_	1	1.0267	0.8440	0.8791	0.8251	0.7258	0.7266	0.6877	0.7629	0.7566	0.7128	0.6926	0.8705	0.5659
x_1_	1	1.0630	0.8740	1.0000	1.1339	1.1811	0.9764	0.9764	0.9449	0.9055	0.8898	0.8898	0.8245	0.8984
x_2_	1	1.0315	1.0320	1.3264	1.1481	1.2775	1.2900	1.2627	1.4608	1.2130	0.1935	0.1958	1.3870	1.4782
x_3_	1	0.9694	0.9425	1.0371	1.3303	1.4156	1.5289	1.6418	1.8926	1.7879	0.5733	0.5539	1.7283	1.5846
x_4_	1	0.8004	1.1006	0.9444	0.8012	0.3554	0.3422	0.2805	0.2381	0.2716	0.8532	0.8637	0.6257	0.7105
x_5_	1	1.1547	1.3328	1.0842	0.9229	0.9062	0.7804	0.9725	1.3592	1.3493	1.3633	1.4408	1.2586	1.3411
x_6_	1	1.1710	1.0251	1.0454	1.3819	1.3245	1.5937	1.2356	0.9137	0.9837	1.3651	1.3434	1.1447	1.2291
x_7_	1	1.0317	1.0291	1.0305	1.0167	1.0009	0.9880	0.9428	0.9149	0.8832	0.8603	0.8462	0.8413	0.8345

**Table 6:** Calculation results of differential sequences through Grey correlation analysis (2003–2016).

Diff. Seq.	2003	2004	2005	2006	2007	2008	2009	2010	2011	2012	2013	2014	2015	2016
x_0_-x_1_	0	0.0363	0.0300	0.1209	0.3088	0.4553	0.2498	0.2887	0.1819	0.1489	0.1770	0.1972	0.0459	0.3325
x_0_-x_2_	0	0.0047	0.1880	0.4473	0.3231	0.5517	0.5633	0.5750	0.6979	0.4564	0.5193	0.4968	0.5166	0.9123
x_0_-x_3_	0	0.0574	0.0985	0.1581	0.5053	0.6898	0.8023	0.9541	1.1296	1.0313	0.1394	0.1386	0.8578	1.0187
x_0_-x_4_	0	0.2264	0.2566	0.0653	0.0238	0.3704	0.3845	0.4072	0.5248	0.4850	0.1404	0.1712	0.2447	0.1446
x_0_-x_5_	0	0.1280	0.4888	0.2051	0.0978	0.1804	0.0538	0.2848	0.5962	0.5928	0.6506	0.7482	0.3881	0.7752
x_0_-x_6_	0	0.1442	0.1811	0.1663	0.5568	0.5987	0.8671	0.5479	0.1507	0.2271	0.6524	0.6508	0.2742	0.6632
x_0_-x_7_	0	0.0050	0.1851	0.1514	0.1917	0.2751	0.2614	0.2551	0.1519	0.1266	0.1475	0.1536	0.0292	0.2686

**Table 7:** Results of Grey correlation (2003–2016).

Variable	2003	2004	2005	2006	2007	2008	2009	2010	2011	2012	2013	2014	2015	2016
x_1_	1	0.9397	0.9496	0.8237	0.6465	0.5537	0.6934	0.6618	0.7564	0.7913	0.7614	0.7412	0.9248	0.6295
x_2_	1	0.9917	0.7503	0.5580	0.6361	0.5059	0.5007	0.4955	0.4473	0.5531	0.5210	0.5320	0.5223	0.3824
x_3_	1	0.9078	0.8516	0.7814	0.5278	0.4502	0.4132	0.3719	0.3333	0.3539	0.8020	0.8029	0.3970	0.3567
x_4_	1	0.7139	0.6876	0.8964	0.9595	0.6040	0.5950	0.5811	0.5184	0.5380	0.8009	0.7674	0.6977	0.7962
x_5_	1	0.8153	0.5361	0.7336	0.8524	0.7579	0.9131	0.6648	0.4865	0.4879	0.4647	0.4302	0.5927	0.4215
x_6_	1	0.7966	0.7572	0.7725	0.5036	0.4854	0.3944	0.5076	0.7894	0.7132	0.4640	0.4646	0.6732	0.4599
x_7_	1	0.9913	0.7532	0.7886	0.7466	0.6725	0.6836	0.6889	0.7880	0.8169	0.7930	0.7862	0.9508	0.6777

**Table 8:** Grey correlations and ranks between intra-regional difference of provincial efficiency difference of Chinese township hospitals and the determinants.

Correlation	*γ*_01_	*γ*_02_	*γ*_03_	*γ*_04_	*γ*_05_	*γ*_06_	*γ*_07_
Value	0.7766	0.5997	0.5964	0.7254	0.654	0.6273	0.7955
Rank	2	6	7	3	4	5	1

**Table 9:** Grey correlations and ranks between intra-regional difference of provincial efficiency difference of Chinese township hospitals and the determinants in each region.

Correlation	Eastern		Central		Western	
	Value	Rank	Value	Rank	Value	Rank
***γ*_01_**	0.6682	5	0.8068	3	0.8475	3
***γ*_02_**	0.6187	6	0.5063	7	0.8488	2
***γ*_03_**	0.7484	4	0.8191	2	0.6184	7
***γ*_04_**	0.8803	1	0.6448	6	0.8079	4
***γ*_05_**	0.7784	3	0.7588	5	0.735	5
***γ*_06_**	0.5675	7	0.7822	4	0.8755	1
***γ*_07_**	0.833	2	0.8795	1	0.7332	6
